# Traumatic Bereavement Among Incarcerated Women: Understanding and Responding to Complex Grief After the Death of a Child

**DOI:** 10.3390/bs16091573

**Published:** 2026-09-04

**Authors:** Catherine Gallagher, Nena P. Messina

**Affiliations:** 1 Criminology, Law and Society, George Mason University, Fairfax, VA 22030, USA; 2 Envisioning Justice Solutions, Simi Valley, CA 93065, USA; nena@envisioningjusticesolutions.com

**Keywords:** traumatic bereavement, incarcerated women, maternal grief, child death, meaning-making, correctional mental health, social determinants of health, substance use, resilience

## Abstract

Incarcerated women face elevated suicide risk at the intersection of chronic loss, cumulative trauma, and constrained environments. In prison, the death of a child constitutes a profound and overlooked traumatic bereavement. This conceptual manuscript examines how carceral environments restrict privacy, autonomy, family contact, mourning rituals, and social and clinical support, thereby adversely reshaping grief. The Dual Process Model of Coping and meaning-making frameworks are integrated to explain how incarceration may constrain oscillation between loss-oriented and restoration-oriented coping. Institutional functioning may resemble restoration-oriented behavior while lacking critical adaptive factors that define restorative coping. Restricted information, exclusion from rituals and relational validation, and punishment for grief-related behavior may obstruct narrative reconstruction, continuing bonds, and maternal identity reorganization. The model further considers how acute grief interacts with chronic stress, sustained threat responsivity, sleep disruption, and cumulative allostatic burden to intensify psychological and somatic distress without assuming a uniform neurobiological pathway. Social determinants of health, histories of victimization, substance use, and facility-level conditions are important sources of variation. Protective pathways are also specified, including family connection, peer support, cultural and spiritual practices, and clinical care. The paper concludes with testable propositions, a research agenda, and a framework for notification, stabilization, and continuing bereavement support.

## 1. Introduction

### 1.1. The Intersection of Incarceration, Maternal Status, and Traumatic Bereavement

Maternal identity remains central for many incarcerated women, even when legal processes, geographic distance, restricted communication, and disrupted caregiving have made the parent–child relationship difficult to sustain. Separation from children may already be accompanied by guilt, shame, uncertainty, and grief (Rodríguez-Lirón et al., 2024). Within this baseline of instability, the death of a child can constitute an acutely destabilizing loss that is intensified by physical confinement and exclusion from ordinary family and community responses (Harner et al., 2011; McLean et al., 2024).

Available accounts suggest that institutional practices for notifying incarcerated parents of a child’s death vary substantially and may not consistently provide privacy, trained support, verified information, or immediate family contact (McLean et al., 2024). A mother may receive brief or incomplete notice and then be expected to return to routine housing, work, movement, and behavioral requirements. For women who enter custody with extensive histories of victimization, mental health difficulties, substance use, family disruption, and prior loss, these conditions may compound the original bereavement and impede adaptation (Hopf et al., 2020).

The central argument of this paper is that the loss of a child from sudden, traumatic causes, as well as the loss of a child while separated in custody, may result in traumatic bereavement. Further, grief is not simply carried into prison. It is actively shaped by the conditions under which the loss is learned, understood, expressed, and socially recognized. The same institution may intensify risk through isolation, punishment, and blocked communication or buffer harm through timely information, relational connection, protected mourning, and clinically responsive care (Messina & Covington, 2026).

### 1.2. Conceptual Boundaries and Definitions

This manuscript uses the term complex grief descriptively to refer to traumatic bereavement made more difficult by traumatic circumstances, cumulative adversity, disrupted relationships, and environmental constraint. The term is not used as a diagnosis and does not imply that intense parental grief is inherently pathological. Prolonged grief disorder, post-traumatic stress disorder (PTSD), depression, and expected acute grief are related but distinct outcomes and should be assessed separately in empirical work (Szuhany et al., 2021).

Traumatic bereavement is conceptualized as the sudden, violent, or unexpected death of a close other, as well as a loss that becomes traumatic because of the circumstances in which it is experienced (Ennis et al., 2023; Solomon, 2024). In custody, abrupt or impersonal notification, incomplete or unverified information, enforced absence, inability to see the child or participate in rituals, and uncertainty about what occurred may render even an anticipated death acutely traumatic (Solomon, 2024).

For clinical and research purposes, the term mother refers to people who identify with or have occupied a primary maternal caregiving role. This includes biological, adoptive, step, foster, kinship, and gender-diverse parents or caregivers. Relationships with the deceased child may have been close, disrupted, estranged, or ambivalent; these differences should not be collapsed in research or clinical assessment.

Some women may have lost formal or informal caregiving roles because of substance use, criminal involvement, child welfare intervention, incarceration, or other circumstances. Conversely, separation from an incarcerated mother may at times be experienced by children or other family members as protective or stabilizing when separation reduces exposure to pre-incarceration instability, violence, or other harms. Yet, this framework contends that maternal identity may remain psychologically central even when the mother does not have formal custody or the child’s day-to-day care is provided by someone else. Lack of custody or daily caregiving does not necessarily alter how a woman understands her identity as a mother.

## 2. The Carceral Environment as a Modifier of Bereavement

### 2.1. Epidemiological Reality and the Gendered Gap

Over the past four decades, the population of incarcerated women in the United States has increased by approximately 700%, growing from 26,000 in 1980 to over 230,000 women in prisons and jails today (Carson & Kluckow, 2023). Within this invisible and highly vulnerable demographic, approximately 58% identify as mothers of minor children (Messina, 2021). Although the precise population fluctuates across years and data systems, women in custody remain poorly represented in bereavement research and in correctional policies addressing the death of a child.

Women in custody experience high rates of trauma, victimization, substance-use disorders, poverty, and family disruption (Lynch et al., 2014; Messina et al., 2020; Messina & Grella, 2006; Owen et al., 2017). Recent data from a sample of 5775 women in prison characterize trauma, psychological distress, and addiction as nearly ubiquitous, underscoring the clinical complexity of this population (Carei et al., 2025). Empirical assessments of this population demonstrate that over 60% have been previously diagnosed with a mental illness, and they carry an average of four out of ten adverse childhood experiences (ACEs) (Messina & Grella, 2006; Messina & Schepps, 2021). Specifically, over half have survived childhood physical (54%) or sexual abuse (51%), while adult victimization rates are exceptionally high, with 79% experiencing physical abuse and 73% experiencing severe intimidation in adulthood (Saxena & Messina, 2021).

These experiences should not be treated as interchangeable or as fixed individual liabilities. They are partly produced by upstream social determinants of health and may interact with the circumstances of the child’s death, the quality of the parent–child relationship, time since loss, sentence characteristics, and the institutional response (Alderwick & Gottlieb, 2019; Harner et al., 2011). These complex traumatic histories and disrupted relationships may also shape grief responses and increase sensitivity to institutional conditions such as surveillance, restricted autonomy, limited privacy, and disrupted social support. These characteristics may represent both baseline vulnerabilities and potential moderators of how carceral grief conditions influence coping, meaning-making, and psychological distress. These differences support individualized, trauma-informed bereavement care, including targeted support for women with extensive trauma histories (Messina & Covington, 2026; Messina & Esparza, 2022) and potentially specialized support for those bereaved by suicide.

The cause of death may alter the content and processes of grieving without determining a uniform trajectory. Death by suicide may introduce challenges related to guilt, perceived maternal responsibility, unanswered questions, stigma, and heightened maternal suicide risk, whereas illness and other causes of death may involve different anticipatory, relational, or meaning-making processes (Hendrickson, 2009; Pitman et al., 2014). Certain studies theorize that mothers bereaved by suicide may also experience a sense of failure for not preventing their child’s death or fulfilling their protective role (Hendrickson, 2009).

Across jurisdictions, incarcerated women experience markedly higher suicide rates than women in the general population, with a gender disparity that is often greater than the corresponding disparity among incarcerated men (Dye, 2011; Mennicke et al., 2021; Mundt et al., 2024). Asphyxiation is a common method of suicide in custody among women, underscoring the importance of environmental safety and timely clinical assessment (Bardale & Dixit, 2015; Gallagher & Dobrin, 2006a). Method patterns, however, should not be interpreted as evidence that bereaved women uniformly intend to evade surveillance or that a single pathway explains suicidal behavior.

Correctional suicide-prevention systems have historically emphasized intake screening, intoxication, cell design, ligature hazards, and male patterns of risk (Gallagher & Dobrin, 2005, 2006b, 2007; Hayes, 2012). These components remain important, but they may overlook pathways that are especially salient for women, including cumulative interpersonal trauma, carceral trauma, maternal separation, family loss, shame, substance-use histories, and the institutional consequences of expressing distress (Favril & Vander Laenen, 2019; Fedock et al., 2021).

### 2.2. Compounded Loss and Rigid Architecture

Prisons and jails are designed to maximize control, surveillance, predictability of movement, and operational standardization. These features can conflict with privacy, relational safety, autonomy, and flexibility that support mourning. Confinement may separate a mother from already fragile support networks, restrict access to information about the child’s death, limit participation in funeral or memorial practices, and increase observation during a period of acute vulnerability (Fedock et al., 2021; Harner et al., 2011).

These conditions may simultaneously intensify loss-oriented stressors and impose mandatory functional demands. In the community, restoration-oriented coping generally involves voluntary and self-paced engagement with altered roles and daily life (Stroebe & Schut, 2010; Larsen et al., 2025). In custody, outward functioning may instead reflect compliance with counts, work assignments, movement schedules, program requirements, and security rules. Forced institutional functioning may therefore resemble restoration behaviorally without providing the autonomy or adaptive purpose that makes restoration psychologically useful.

The carceral environment may constrain adaptation through several interrelated mechanisms:

- Restricted control and physical escape: A grieving parent in the community may seek privacy, move between settings, visit family, or temporarily withdraw from demands. An incarcerated mother has far less control over location, timing, companionship, and sensory input, which may reinforce helplessness and separation.
- Sensory intrusiveness and limited privacy: Housing units may include unpredictable noise, loud announcements, clanging doors, crowding, distinct odors, nighttime disruption, and frequent observation. During acute grief, these conditions may heighten arousal, interfere with sleep and concentration, and reduce opportunities for emotional regulation.
- Surveillance and suppression of affect: Crying, panic, withdrawal, refusal to eat, agitation, or a need for solitude may occur under scrutiny from staff and peers. When women believe that visible distress will trigger disciplinary consequences, loss of privileges, or restrictive housing, they may mask symptoms and avoid seeking help.
- Restricted information, ritual, and continuing bonds: Delayed communication, inability to speak at length with relatives, exclusion from funerals, and limited access to photographs, belongings, spiritual practices, or memorial activities may impede reality confirmation, shared mourning, and the reconstruction of an ongoing bond with the deceased child.

### 2.3. Security-Driven Responses and the Repression of Mourning

Because correctional settings are organized primarily around custody and operations rather than bereavement care, expected grief reactions may be interpreted as noncompliance, behavioral disruption, or evidence of acute security risk. This does not mean that all facilities respond identically or that all safety interventions are inappropriate. It does mean that grief-specific assessment is needed before an institution equates distress with misconduct or automatically applies its most restrictive response. The framework therefore calls for grief-informed staff training to distinguish acute grief reactions from behaviors indicating imminent safety or security concerns, while recognizing that the two may coexist, and to support proportionate responses that maintain necessary security measures without unnecessary punishment or isolation.

Depending on the facility and circumstances, security-driven responses may include the following:

- Removal of possessions or comfort items;
- Restriction of telephone, video, or visitation access;
- Interruption of work, programming, or peer contact without a clinical plan;
- Placement in restrictive housing, protective custody, or a suicide-watch setting.

Restrictive isolation is often used as a means of suicide prevention (Fedock et al., 2021). Isolation can remove relational supports, worsen disorientation, and discourage honest disclosure when women fear that requesting help will lead to further deprivation (Augustine et al., 2021). Suicide precautions should follow individualized clinical assessment and use the least-restrictive, most relationally supportive setting compatible with immediate safety. Implementation of least-restrictive approaches to suicide prevention may be difficult in facilities with limited staffing, mental health resources, and specialized training. The framework therefore recognizes that relational and individualized approaches must be adapted to facility capacity while maintaining immediate safety.

Peer dynamics are similarly complex. Other incarcerated women may provide rapid recognition, practical support, prayer, companionship, or protection that the formal institution does not supply (Covington, 2024). At the same time, institutional norms may pressure a bereaved mother to limit visible grief, resume routine, or avoid appearing vulnerable. Peer culture should therefore be studied as a potential source of both protection and constraint rather than characterized as uniformly supportive or punitive.

### 2.4. The Gendered Burden of Enforced Maternal Rupture

Child loss is filtered through social and familial expectations surrounding motherhood. Justice-involved mothers may already be blamed for their absence, judged as undeserving of maternal status, or excluded from family decision-making. After a child’s death, these external judgments may intensify guilt, shame, anger, helplessness, sense of failure, and fears that they have forfeited the right to grieve (Rodríguez-Lirón et al., 2024).

The loss may therefore destabilize not only attachment to the child but also the mother’s sense of identity, purpose, and relational continuity (Neimeyer, 2011). The death may represent a final rupture in the maternal relationship and the irreversible loss of any opportunity to repair or restore that relationship, intensifying suicidal distress. This rupture occurs in an environment that often rewards emotional control and rapid return to routine. The resulting conflict may strain coping resources, increase isolation, and complicate the reconstruction of a maternal identity, even among surviving children.

Traditional accounts of resilience are inadequate when they imply a return to a pre-loss baseline (Bonanno & Westphal, 2024). Parental bereavement may require enduring identity transformation rather than recovery of a prior state (Neimeyer, 2011). This paper proposes the term inhibited resilience to describe circumstances in which adaptive capacities remain present but cannot be fully enacted because the environment restricts autonomy, emotional expression, relational access, ritual, and opportunities for meaningful reconstruction. Inhibited resilience is a provisional conceptual construct, not a diagnosis, fixed trait, or synonym for symptom severity. Its value depends on demonstrating that the prison environment blocks the expression of coping capacities that are otherwise available.

## 3. A Conceptual Model of Carceral Bereavement

The proposed model treats bereavement in custody as the interaction of four domains: (1) baseline and relational context; (2) institutional grief conditions; (3) psychological, relational, and physiological mechanisms; and (4) deleterious and adaptive outcomes. Baseline context includes prior trauma, mental health and substance-use histories, social position, sentence and facility characteristics, the nature of the maternal relationship, the cause and anticipatedness of death, and time since loss. Institutional grief conditions include how notification occurs, access to verified information and family, participation in rituals, privacy, autonomy, staff and peer responses, and the use of restrictive security practices.

### 3.1. Carceral Constriction of the Dual Process Model of Coping

The Dual Process Model (DPM) of Coping conceptualizes adaptation to bereavement as movement between loss-oriented and restoration-oriented stressors (Stroebe & Schut, 1999, 2010). Loss-oriented coping involves confronting the reality and emotional consequences of the death, while restoration-oriented coping involves attending to changed roles, daily tasks, and temporary respite from grief. Oscillation does not require equal time in each orientation and is not a linear progression (Larsen et al., 2025).

Custody may constrain both sides of this process. Privacy, emotional expression, ritual, and extended family conversation may be unavailable, limiting loss-oriented processing. At the same time, work assignments, counts, movement, and behavioral rules compel outward functioning before the mother has had an opportunity to understand or integrate the loss. This paper distinguishes forced institutional functioning from restoration-oriented coping. Compliance may protect a woman from disciplinary consequences, but it should not be treated as evidence that she has adapted, achieved respite, or regained psychological capacity.

The degree of constriction is expected to vary. Even within highly controlled settings, access to a trusted peer, a private call, a chaplain, a meaningful object, or a flexible staff response may create limited opportunities for oscillation. The model therefore treats institutional conditions as measurable exposures rather than assuming that all incarcerated mothers experience an identical grief environment.

### 3.2. Institutional Disruptions to Meaning-Making

Meaning-making frameworks distinguish between global meaning—core beliefs about identity, purpose, predictability, fairness, and self-worth—and the situational meaning assigned to a particular event (Neimeyer et al., 2008). A child’s death can produce a profound discrepancy between these systems. Adaptation may involve understanding what occurred, revising assumptions, reconstructing a coherent narrative, preserving a continuing bond, and locating a viable identity after the loss.

Carceral conditions may obstruct these processes when the mother receives incomplete information, cannot speak extensively with witnesses or relatives, is excluded from funeral and memorial practices, or lacks culturally meaningful ways to acknowledge her child. Under these conditions, uncertainty and relational alienation may sustain rumination, disbelief, guilt, or a sense that the death remains psychologically unreal. The global meaning system does not inevitably collapse, but the institution may deprive the mother of important resources for reducing discrepancy and reconstructing purposes.

Conversely, family narratives, photographs, letters, memorial objects, spiritual or cultural rituals, and opportunities to speak about the child may support reality integration and continuing bonds (Hynson et al., 2006; Lichtenthal et al., 2010). Meaning reconstruction should therefore be treated as a relational and institutional process, not solely an intrapsychic task assigned to the bereaved mother.

### 3.3. Inhibited Resilience and Protective Pathways

Resilience is best understood as a dynamic process produced through transactions between the person and the environment. Cognitive flexibility, hope, active coping, spirituality, humor, relational trust, and the ability to seek support may exist within the individual, but their expression depends on opportunity. A mother cannot use family connection as a coping resource when calls are delayed; she cannot engage in private ritual when no private space exists; and she may not disclose distress when disclosure is expected to produce isolation (Harner et al., 2011).

Inhibited resilience describes this gap between capacity and enactment. The model predicts that highly restrictive grief conditions will weaken the association between pre-existing resilience resources and adaptive outcomes. It also predicts that even modest changes in the environment may release capacities that were obscured by institutional constraint.

Protective pathways may include timely contact with surviving family, trained and supervised peer support, validating staff responses, access to chaplaincy or culturally congruent ritual, opportunities to create memorials, continuing bonds with the child, engagement in helping roles, and reconstruction of maternal identity through memory, legacy, or care for surviving children and community. These pathways do not guarantee post-traumatic growth or eliminate severe distress. They provide conditions under which adaptation, connection, and psychological continuity become more possible.

### 3.4. Stress-System Burden and Somatic Dysregulation

Traumatic loss can produce heightened arousal, intrusive thoughts, sleep disruption, autonomic activation, and changes in attention and emotion regulation (Hopf et al., 2020). Chronic confinement, unpredictability, sensory load, and perceived threat may prolong or repeatedly reactivate these responses. Neurobiological models suggest that systems involved in threat processing, emotional regulation, autonomic functioning, and stress responses may be affected (Ressler et al., 2022). The available evidence does not support the assumption that these systems uniformly fail, become permanently locked, or progress through a single sequence in every bereaved woman (Bonanno et al., 2023; Donaldson & Shear, 2024).

A more defensible formulation is that acute bereavement and chronic institutional stress may contribute to stress-system dysregulation and cumulative allostatic burden, reflecting the physiological costs of repeated or sustained stress (Guidi et al., 2021). Observable manifestations may include persistent or recurrent hyperarousal, sleep disturbance, gastrointestinal symptoms, altered pain, fatigue, concentration difficulties, and cardiovascular strain. These symptoms may affect behavior and coping, but they should not automatically be interpreted as misconduct, proof of neuroendocrine exhaustion, or a direct cause of suicidal behavior.

PTSD, prolonged grief disorder, depression, suicidal distress, and somatic symptoms may co-occur, but none should be inferred solely from the setting or from visible grief. Empirical studies should assess these outcomes separately and examine whether institutional conditions predict change over time. Biomarker evidence, including salivary cortisol measures, has been examined in female prison populations (Brewer-Smyth et al., 2004), but direct biomarker or neuroimaging evidence among incarcerated mothers grieving a child is currently limited. Physiological mechanisms should therefore be presented as testable pathways rather than established causal facts.

### 3.5. Research Propositions

The model generates six testable propositions for future empirical evaluation. These propositions are intended to guide research design rather than specify a single measurement strategy or causal pathway:

**Proposition** **1.** *Greater restriction in institutional grief conditions—including delayed notification, inadequate information, restricted family contact, lack of privacy, and exclusion from ritual—will be associated with higher grief-related distress, trauma symptoms, somatic burden, and suicidal ideation after accounting for baseline differences.*

**Proposition** **2.** *Forced institutional functioning will not predict adaptive restoration unless it is accompanied by some degree of autonomy, psychological respite, and opportunity for loss-oriented processing.*

**Proposition** **3.** *Disruption of meaning-making and maternal identity reconstruction will partially mediate associations between constrictive institutional conditions and subsequent distress.*

**Proposition** **4.** *Relational connection, culturally meaningful ritual, peer and staff validation, and access to continuing bonds will buffer adverse psychological and somatic outcomes and support adaptive continuity.*

**Proposition** **5.** *Punitive or automatically restrictive responses to grief behavior will be associated with worsening distress, concealment of symptoms, and suicidal risk, whereas individualized least-restrictive care will attenuate these associations.*

**Proposition** **6.** *Facility-level differences—including jail versus prison setting, security classification, staffing, communication rules, and local bereavement policies—will explain variation in outcomes beyond individual characteristics.*

## 4. Methodological Blueprints, Testable Hypotheses, and Interventions

The conceptual propositions can be translated into feasible empirical models and provisional practice recommendations. The goal is not to infer a single biological pathway or to treat all grief as pathology, but to identify modifiable institutional exposures, mechanisms, and protective conditions.

### 4.1. Feasible Empirical Hypotheses

**Hypothesis** **1 (Institutional Constriction).** *Among bereaved mothers in custody, greater restriction of privacy, information, family contact, ritual, and schedule flexibility will predict higher grief-related distress and trauma symptoms over time, controlling for baseline trauma, mental health, substance use, relationship characteristics, cause of death, and time since loss.*

**Hypothesis** **2 (Meaning-Making Mediation).** *Difficulty obtaining verified information, participating in memorial practices, and reconstructing maternal identity will mediate the association between restrictive institutional conditions and subsequent grief-related distress.*

**Hypothesis** **3 (Autonomy and Relational Buffering).** *Perceived autonomy, family communication, peer support, staff validation, and cultural or spiritual continuity will moderate the effects of forced institutional functioning, such that stronger supports are associated with greater adaptive coping and lower distress.*

**Hypothesis** **4 (Institutional Response and Acute Risk).** *Punitive or automatically restrictive responses to visible grief will predict short-term increases in concealment, hopelessness, behavioral dysregulation, and suicidal ideation, whereas individualized least-restrictive clinical care will predict stabilization and help-seeking.*

**Hypothesis** **5 (Somatic Burden).** *Greater exposure to constrictive grief conditions will be associated with sleep disturbance, hyperarousal, perceived stress, pain, gastrointestinal symptoms, and fatigue. Subsequent biomarker studies may evaluate whether these associations are accompanied by alterations in stress-system functioning.*

### 4.2. Opportunities and Provisional Operational Timeframes

The following windows are theory-informed practice periods rather than empirically validated cutoffs. The pace and intensity of support should be individualized according to the circumstances of the death, current symptoms, safety, cultural practices, family access, and the mother’s preferences.

1. The Notification Window (initial hours and first day): Notification should be delivered privately by a trained mental health professional or designated staff member with bereavement training whenever feasible. When one is unavailable, trained correctional or peer mentors could provide initial notification and support according to a standardized bereavement protocol, with prompt clinical referral when indicated. The mother should receive clear, verified information; an opportunity to ask questions; and prompt, private, and sufficiently extended contact with chosen family or another chosen support. Acute grief reactions should not automatically result in restrictive isolation. Suicide precautions should follow individualized assessment and use the least restrictive, most relationally supportive setting consistent with immediate safety.

2. The Stabilization Window (early days and weeks): Facilities should provide repeated clinical contact, reasonable temporary flexibility in work, programming, and movement, access to a quieter or more private setting when safe, support for sleep and somatic symptoms, and continued family, peer, cultural, or spiritual connection. Support should be revisited rather than withdrawn according to a fixed timetable.

3. The Continuity Window (subsequent months and as needed): Longer-term care should address meaning reconstruction, continuing bonds, maternal identity, relationships with surviving family, anniversaries, legal or family developments, and grief-specific treatment when clinically indicated. Peer and staff support should remain available without imposing either an expectation of rapid recovery or a requirement that the woman display visible distress to qualify for care.

### 4.3. Operationalizing a Carceral Bereavement-Care Framework

The staged approach can be translated into seven institutional domains:

- Dedicated bereavement protocols: Create a written, multidisciplinary pathway that begins when notice of a child’s serious illness or death is received and specifies notification, documentation, clinical assessment, family contact, property access, accommodations, and follow-up.
- Protected mourning: Provide reasonable temporal and physical accommodations for grief expression while preserving safety. Temporary flexibility should not be framed as exemption from all behavioral expectations, and visible grief should not be treated as misconduct.
- Family connection: Provide timely, private, and minimally restricted telephone or video contact with surviving relatives or another chosen support, subject to individualized and necessary safety limitations.
- Ritual, cultural, and spiritual continuity: Facilitate culturally and spiritually meaningful mourning practices, including memorial rituals, chaplaincy, photographs, letters, memory objects, and other forms of continuing bond, when safely feasible. Designated memorial spaces or gardens could provide opportunities for bereaved mothers to participate in maintaining memorials for their children, consistent with facility security and operational requirements.
- Peer support networks: Develop voluntary, trained, supervised, and supported peer roles, including potential peer grief navigators and, where feasible, specialized peer support for suicide bereavement and other high-risk grief circumstances. Peer supporters should not replace clinicians or bear unbounded responsibility for crisis management.
- Staff training: Train correctional, medical, and mental health staff to distinguish expected acute grief, trauma symptoms, clinical deterioration, and security concerns and to respond without unnecessary punishment or deprivation. Staff training should support recognition of culturally specific mourning practices and individualized accommodation within necessary security and operational constraints.
- Clinically responsive and least-restrictive care: Use individualized assessment, repeated follow-up, contemporary grief and trauma measures, and evidence-informed treatment. Monitoring should be relational and therapeutic rather than solely observational or mechanical.

## 5. Conclusions: From Punitive Control to Clinically Responsive Care

Maternal incarceration and the death of a child intersect within an institution that can either intensify or reduce the harms of traumatic bereavement. The carceral environment is not a neutral backdrop. Notification practices, access to information and family, privacy, ritual, staff and peer responses, and the use of restrictive security measures shape whether a mother can confront the loss, obtain respite, reconstruct meaning, and enact available coping capacities.

The central theoretical contribution is the distinction between restoration-oriented coping and forced institutional functioning. Behavioral compliance may be necessary for survival in custody, but it should not be mistaken for adaptation. When loss-oriented processing is restricted and outward functioning is compelled, the oscillation described by the DPM may be narrowed. Meaning-making and resilience are likewise dependent on relational and institutional resources, not solely on individual strength.

A clinically responsive approach does not require institutions to abandon safety. It requires individualized assessment, least-restrictive care, timely family contact, protected mourning, cultural and spiritual continuity, trained peer and staff support, and sustained follow-up. These practices remain theory-informed and require evaluation, but they identify modifiable conditions that can be studied and improved. At the policy level, implementation would require correctional systems to incorporate bereavement response into formal policy, staff training requirements, and resource planning. Scalable implementation should prioritize standardized protocols, grief-informed staff and peer training, and flexible mechanisms for family contact, clinical referral, and culturally responsive mourning within existing safety and operational constraints.

Future research should measure institutional grief conditions directly, separate grief from PTSD and other outcomes, account for facility-level variation, and examine both deleterious and adaptive pathways. Doing so is necessary to understand how incarcerated mothers survive the death of a child and to ensure that natural mourning is met with dignity, relational support, and clinically appropriate care.

## Data Availability

No new data were created or analyzed in this study. Data sharing is not applicable to this article.
